# Supplementary material for: Most primary olfactory neurons have individually neutral effects on behavior
Source: eLife. 2022 Jan 19;11:e71238. doi: 10.7554/eLife.71238 (PMC8806191; doi:10.7554/eLife.71238)
Supplement: Supplementary file 1. — The innate responses of 24 types of ORNs that have been reported at time of writing (January, 2020). The valence response, experimental assay, stimulus type, sex, and the developmental stage of the flies varied across studies. The valence column shows the response direction produced by the respective ORN: negative (-), positive (+), or indifferent (o). The assay column presents the nature of the assay used in the experiments: oviposition (place preference for egg-laying in female flies), two-choice (any assay by which the flies are presented with a choice to activate the ORN), locomotor (the assay in which motor behavior of the larvae is used to deduce valence). The stimulus column shows the type of the stimulus applied on the ORN: olfactogenetics (geosmin on ORNs that ectopically express the Or56a receptor), optogenetic (light stimulus on genetically modified ORNs), odor. In the sex column, F, M, M/F, and N/A indicate female, male, both male and female, and information that is not available, respectively. The stage column presents the developmental stage of the animals used in the experiment: larva, adult, and both (larva and adult). [file elife-71238-supp1.docx]

### Supplementary File 1. ORN types with known innate valence.

The innate responses of 24 types of ORNs that have been reported at time of writing (January, 2020). The valence response, experimental assay, stimulus type, sex, and the developmental stage of the flies varied across studies. The valence column shows the response direction produced by the respective ORN: negative (-), positive (+), or indifferent (o). The assay column presents the nature of the assay used in the experiments: oviposition (place preference for egg-laying in female flies), two-choice (any assay by which the flies are presented with a choice to activate the ORN), locomotor (the assay in which motor behavior of the larvae is used to deduce valence). The stimulus column shows the type of the stimulus applied on the ORN: olfactogenetics (geosmin on ORNs that ectopically express the Or56a receptor), optogenetic (light stimulus on genetically modified ORNs), odor. In the sex column, F, M, M/F, and N/A indicate female, male, both male and female, and information that is not available, respectively. The stage column presents the developmental stage of the animals used in the experiment: larva, adult, and both (larva and adult).

| **Receptor** | **Valence** | **Assay** | **Stimulus** | **Sex** | **Stage** | **Reference** |
| --- | --- | --- | --- | --- | --- | --- |
| Or7a | – | oviposition | olfactogenetics | F | adult | [(Chin et al., 2018)](https://paperpile.com/c/yPRhVL/Zm1rP) |
| Or7a | o | two-choice | optogenetic | M | adult | ***this study*** |
| Or19a | + | oviposition | odor | F | adult | [(Dweck et al., 2013)](https://paperpile.com/c/yPRhVL/gr6sq) |
| Or19a | o | oviposition | olfactogenetics | F | adult | [(Chin et al., 2018)](https://paperpile.com/c/yPRhVL/Zm1rP) |
| Or19a | o | two-choice | optogenetic | M | adult | ***this study*** |
| Or22a | + | two-choice | odor | M/F | adult | [(Knaden et al., 2012)](https://paperpile.com/c/yPRhVL/qLe93) |
| Or22a | + | two-choice | odor | F | adult | [(Semmelhack and Wang, 2009)](https://paperpile.com/c/yPRhVL/GrbPO) |
| Or22a | – | two-choice | odor | F | adult | [(Gao et al., 2015)](https://paperpile.com/c/yPRhVL/jHkgo) |
| Or22a | + | two-choice | optogenetic | M | adult | [(Bell and Wilson, 2016)](https://paperpile.com/c/yPRhVL/2nWjO) |
| Or22a | o | oviposition | olfactogenetics | F | adult | [(Chin et al., 2018)](https://paperpile.com/c/yPRhVL/Zm1rP) |
| Or22a | o | two-choice | optogenetic | M | adult | ***this study*** |
| Or23a | o | oviposition | olfactogenetics | F | adult | [(Chin et al., 2018)](https://paperpile.com/c/yPRhVL/Zm1rP) |
| Or23a | o | two-choice | optogenetic | M | adult | ***this study*** |
| Or35a | o | oviposition | olfactogenetics | F | adult | [(Chin et al., 2018)](https://paperpile.com/c/yPRhVL/Zm1rP) |
| Or35a | + | two-choice | optogenetic | M | adult | ***this study*** |
| Or42a | + | locomotor | optogenetic | NA | larva | [(Hernandez-Nunez et al., 2015)](https://paperpile.com/c/yPRhVL/2GxlN) |
| Or42a | + | two-choice | odor | NA | larva | [(Mathew et al., 2013)](https://paperpile.com/c/yPRhVL/10Mo9) |
| Or42a | o | two-choice | odor | F | adult | [(Jung et al., 2015)](https://paperpile.com/c/yPRhVL/lArtb) |
| Or42a | + | two-choice | optogenetic | M | adult | [(Bell and Wilson, 2016)](https://paperpile.com/c/yPRhVL/2nWjO) |
| Or42a | o | oviposition | olfactogenetics | F | adult | [(Chin et al., 2018)](https://paperpile.com/c/yPRhVL/Zm1rP) |
| Or42a | o | two-choice | olfactogenetics | F | adult | [(Chin et al., 2018)](https://paperpile.com/c/yPRhVL/Zm1rP) |
| Or42a | o | two-choice | optogenetic | M | adult | ***this study*** |
| Or42b | + | two-choice | odor | F | adult | [(Semmelhack and Wang, 2009)](https://paperpile.com/c/yPRhVL/GrbPO) |
| Or42b | + | two-choice | odor | NA | larva | [(Mathew et al., 2013)](https://paperpile.com/c/yPRhVL/10Mo9) |
| Or42b | + | two-choice | odor | F | adult | [(Gao et al., 2015)](https://paperpile.com/c/yPRhVL/jHkgo) |
| Or42b | o | two-choice | odor | F | adult | [(Jung et al., 2015)](https://paperpile.com/c/yPRhVL/lArtb) |
| Or42b | + | two-choice | optogenetic | M | adult | [(Bell and Wilson, 2016)](https://paperpile.com/c/yPRhVL/2nWjO) |
| Or42b | o | oviposition | olfactogenetics | F | adult | [(Chin et al., 2018)](https://paperpile.com/c/yPRhVL/Zm1rP) |
| Or42b | + | two-choice | optogenetic | M | adult | ***this study*** |
| Or43a | o | oviposition | olfactogenetics | F | adult | [(Chin et al., 2018)](https://paperpile.com/c/yPRhVL/Zm1rP) |
| Or43a | o | two-choice | optogenetic | M | adult | ***this study*** |
| Or47a | o | oviposition | olfactogenetics | F | adult | [(Chin et al., 2018)](https://paperpile.com/c/yPRhVL/Zm1rP) |
| Or47a | + | two-choice | optogenetic | M | adult | ***this study*** |
| Or47b | – | oviposition | olfactogenetics | F | adult | [(Chin et al., 2018)](https://paperpile.com/c/yPRhVL/Zm1rP) |
| Or47b | + | two-choice | optogenetic | M | adult | ***this study*** |
| Or49a | – | oviposition | olfactogenetics | F | adult | [(Chin et al., 2018)](https://paperpile.com/c/yPRhVL/Zm1rP)c |
| Or49a | o | two-choice | optogenetic | M | adult | ***this study*** |
| Or56a | – | two-choice | odor | NA | adult | [(Stensmyr et al., 2012)](https://paperpile.com/c/yPRhVL/N7lt3) |
| Or56a | + | two-choice | optogenetic | M | adult | [(Bell and Wilson, 2016)](https://paperpile.com/c/yPRhVL/2nWjO) |
| Or56a | – | two-choice | optogenetic | M | adult | ***this study*** |
| Or59c | – | oviposition | olfactogenetics | F | adult | [(Chin et al., 2018)](https://paperpile.com/c/yPRhVL/Zm1rP) |
| Or59c | – | two-choice | optogenetic | M | adult | ***this study*** |
| Or65a | o | oviposition | olfactogenetics | F | adult | [(Chin et al., 2018)](https://paperpile.com/c/yPRhVL/Zm1rP) |
| Or65a | o | two-choice | optogenetic | M | adult | ***this study*** |
| Or67b | + | two-choice | odor | NA | larva | [(Mathew et al., 2013)](https://paperpile.com/c/yPRhVL/10Mo9) |
| Or67b | + | two-choice | optogenetic | M | adult | [(Bell and Wilson, 2016)](https://paperpile.com/c/yPRhVL/2nWjO) |
| Or67b | – | oviposition | olfactogenetics | F | adult | [(Chin et al., 2018)](https://paperpile.com/c/yPRhVL/Zm1rP) |
| Or67b | o | two-choice | optogenetic | M | adult | ***this study*** |
| Or67d | o | oviposition | olfactogenetics | F | adult | [(Chin et al., 2018)](https://paperpile.com/c/yPRhVL/Zm1rP) |
| Or67d | + | two-choice | optogenetic | M | adult | ***this study*** |
| Or71a | – | oviposition | olfactogenetics | F | adult | [(Chin et al., 2018)](https://paperpile.com/c/yPRhVL/Zm1rP) |
| Or71a | o | two-choice | olfactogenetics | F | adult | [(Chin et al., 2018)](https://paperpile.com/c/yPRhVL/Zm1rP) |
| Or71a | o | two-choice | optogenetic | M | adult | ***this study*** |
| Or82a | – | oviposition | olfactogenetics | F | adult | [(Chin et al., 2018)](https://paperpile.com/c/yPRhVL/Zm1rP) |
| Or82a | o | two-choice | optogenetic | M | adult | ***this study*** |
| Or83c | + | two-choice | odor | M/F | adult | [(Ronderos et al., 2014)](https://paperpile.com/c/yPRhVL/aCuyZ) |
| Or83c | – | oviposition | olfactogenetics | F | adult | [(Chin et al., 2018)](https://paperpile.com/c/yPRhVL/Zm1rP) |
| Or83c | + | two-choice | optogenetic | M | adult | ***this study*** |
| Or85a | + | two-choice | odor | M/F | adult | [(Knaden et al., 2012)](https://paperpile.com/c/yPRhVL/qLe93) |
| Or85a | – | two-choice | odor | F | adult | [(Semmelhack and Wang, 2009)](https://paperpile.com/c/yPRhVL/GrbPO) |
| Or85a | – | two-choice | odor | F | adult | [(Gao et al., 2015)](https://paperpile.com/c/yPRhVL/jHkgo) |
| Or85a | + | two-choice | optogenetic | M | adult | [(Bell and Wilson, 2016)](https://paperpile.com/c/yPRhVL/2nWjO) |
| Or85a | – | oviposition | olfactogenetics | F | adult | [(Chin et al., 2018)](https://paperpile.com/c/yPRhVL/Zm1rP) |
| Or85a | o | two-choice | olfactogenetics | F | adult | [(Chin et al., 2018)](https://paperpile.com/c/yPRhVL/Zm1rP) |
| Or85a | o | two-choice | optogenetic | M | adult | ***this study*** |
| Or85d | – | oviposition | olfactogenetics | F | adult | [(Chin et al., 2018)](https://paperpile.com/c/yPRhVL/Zm1rP) |
| Or85d | – | two-choice | optogenetic | M | adult | ***this study*** |
| Or88a | o | oviposition | olfactogenetics | F | adult | [(Chin et al., 2018)](https://paperpile.com/c/yPRhVL/Zm1rP) |
| Or88a | o | two-choice | optogenetic | M | adult | ***this study*** |
| Or92a | + | two-choice | odor | F | adult | [(Semmelhack and Wang, 2009)](https://paperpile.com/c/yPRhVL/GrbPO) |
| Or92a | + | two-choice | optogenetic | M | adult | [(Bell and Wilson, 2016)](https://paperpile.com/c/yPRhVL/2nWjO) |
| Or92a | o | oviposition | olfactogenetics | F | adult | [(Chin et al., 2018)](https://paperpile.com/c/yPRhVL/Zm1rP) |
| Or92a | o | two-choice | optogenetic | M | adult | ***this study*** |
| Gr21a/Gr63a | – | two-choice | odor | NA | adult | [(Suh et al., 2004)](https://paperpile.com/c/yPRhVL/XCKHo) |
| Gr21a/Gr63a | – | two-choice | odor | M/F | both | [(Faucher et al., 2006)](https://paperpile.com/c/yPRhVL/3tp7E) |
| Gr21a/Gr63a | – | two-choice | optogenetic | NA | adult | [(Suh et al., 2007)](https://paperpile.com/c/yPRhVL/hKOAa) |
| Gr21a/Gr63a | – | two-choice | odor | F | adult | [(Poon et al., 2010)](https://paperpile.com/c/yPRhVL/Yotze) |
| Gr21a/Gr63a | – | two-choice | optogenetic | M | adult | [(Bell and Wilson, 2016)](https://paperpile.com/c/yPRhVL/2nWjO) |
| Gr21a/Gr63a | o | oviposition | olfactogenetics | F | adult | [(Chin et al., 2018)](https://paperpile.com/c/yPRhVL/Zm1rP) |
| Gr21a/Gr63a | o | two-choice | olfactogenetics | F | adult | [(Chin et al., 2018)](https://paperpile.com/c/yPRhVL/Zm1rP) |
| Gr21a/Gr63a | – | two-choice | optogenetic | M | adult | ***this study*** |
| Orco | o | two-choice | optogenetic | NA | adult | [*(Suh et al., 2007)*](https://paperpile.com/c/yPRhVL/hKOAa) |
| Orco | + | two-choice | optogenetic | M | adult | [(Bell and Wilson, 2016)](https://paperpile.com/c/yPRhVL/2nWjO) |
| Orco | + | two-choice | optogenetic | M | adult | ***this study*** |

**References**

[Bell JS, Wilson RI. 2016. Behavior Reveals Selective Summation and Max Pooling among Olfactory Processing Channels. *Neuron* **91**:425–438.](http://paperpile.com/b/yPRhVL/2nWjO)

[Chin SG, Maguire SE, Huoviala P, Jefferis GSXE, Potter CJ. 2018. Olfactory Neurons and Brain Centers Directing Oviposition Decisions in Drosophila. *Cell Rep* **24**:1667–1678.](http://paperpile.com/b/yPRhVL/Zm1rP)

[Dweck HKM, Ebrahim SAM, Kromann S, Bown D, Hillbur Y, Sachse S, Hansson BS, Stensmyr MC. 2013. Olfactory preference for egg laying on citrus substrates in Drosophila. *Curr Biol* **23**:2472–2480.](http://paperpile.com/b/yPRhVL/gr6sq)

[Faucher C, Forstreuter M, Hilker M, de Bruyne M. 2006. Behavioral responses of Drosophila to biogenic levels of carbon dioxide depend on life-stage, sex and olfactory context. *J Exp Biol* **209**:2739–2748.](http://paperpile.com/b/yPRhVL/3tp7E)

[Gao XJ, Clandinin TR, Luo L. 2015. Extremely sparse olfactory inputs are sufficient to mediate innate aversion in Drosophila. *PLoS One* **10**:e0125986.](http://paperpile.com/b/yPRhVL/jHkgo)

[Hernandez-Nunez L, Belina J, Klein M, Si G, Claus L, Carlson JR, Samuel AD. 2015. Reverse-correlation analysis of navigation dynamics in Drosophila larva using optogenetics. *Elife* **4**. doi:](http://paperpile.com/b/yPRhVL/2GxlN)[10.7554/eLife.06225](http://dx.doi.org/10.7554/eLife.06225)

[Jung S-H, Hueston C, Bhandawat V. 2015. Odor-identity dependent motor programs underlie behavioral responses to odors. *Elife* **4**. doi:](http://paperpile.com/b/yPRhVL/lArtb)[10.7554/eLife.11092](http://dx.doi.org/10.7554/eLife.11092)

[Knaden M, Strutz A, Ahsan J, Sachse S, Hansson BS. 2012. Spatial representation of odorant valence in an insect brain. *Cell Rep* **1**:392–399.](http://paperpile.com/b/yPRhVL/qLe93)

[Mathew D, Martelli C, Kelley-Swift E, Brusalis C, Gershow M, Samuel ADT, Emonet T, Carlson JR. 2013. Functional diversity among sensory receptors in a Drosophila olfactory circuit. *Proc Natl Acad Sci U S A* **110**:E2134–43.](http://paperpile.com/b/yPRhVL/10Mo9)

[Poon PC, Kuo T-H, Linford NJ, Roman G, Pletcher SD. 2010. Carbon dioxide sensing modulates lifespan and physiology in Drosophila. *PLoS Biol* **8**:e1000356.](http://paperpile.com/b/yPRhVL/Yotze)

[Ronderos DS, Lin C-C, Potter CJ, Smith DP. 2014. Farnesol-detecting olfactory neurons in Drosophila. *J Neurosci* **34**:3959–3968.](http://paperpile.com/b/yPRhVL/aCuyZ)

[Semmelhack JL, Wang JW. 2009. Select Drosophila glomeruli mediate innate olfactory attraction and aversion. *Nature* **459**:218–223.](http://paperpile.com/b/yPRhVL/GrbPO)

[Stensmyr MC, Dweck HKM, Farhan A, Ibba I, Strutz A, Mukunda L, Linz J, Grabe V, Steck K, Lavista-Llanos S, Wicher D, Sachse S, Knaden M, Becher PG, Seki Y, Hansson BS. 2012. A conserved dedicated olfactory circuit for detecting harmful microbes in Drosophila. *Cell* **151**:1345–1357.](http://paperpile.com/b/yPRhVL/N7lt3)

[Suh GSB, 2007. Light Activation of an Innate Olfactory Avoidance Response in Drosophila. *Curr Biol* **17**:905–908.](http://paperpile.com/b/yPRhVL/hKOAa)

[Suh GSB, Wong AM, Hergarden AC, Wang JW, Simon AF, Benzer S, Axel R, Anderson DJ. 2004. A single population of olfactory sensory neurons mediates an innate avoidance behaviour in Drosophila. *Nature* **431**:854–859.](http://paperpile.com/b/yPRhVL/XCKHo)
