# Supplementary material for: Most primary olfactory neurons have individually neutral effects on behavior
Source: eLife. 2022 Jan 19;11:e71238. doi: 10.7554/eLife.71238 (PMC8806191; doi:10.7554/eLife.71238)
Supplement: Transparent reporting form [file elife-71238-transrepform1.docx]

***eLife’s* transparent reporting form**

We encourage authors to provide detailed information *within their submission* to facilitate the interpretation and replication of experiments. Authors can upload supporting documentation to indicate the use of appropriate reporting guidelines for health-related research (see [EQUATOR Network](http://www.equator-network.org/%20)), life science research (see the [BioSharing Information Resource](https://biosharing.org/)), or the [ARRIVE guidelines](http://www.plosbiology.org/article/info:doi/10.1371/journal.pbio.1000412) for reporting work involving animal research. Where applicable, authors should refer to any relevant reporting standards documents in this form.

If you have any questions, please consult our Journal Policies and/or contact us: [editorial@elifesciences.org](mailto:editorial@elifesciences.org).

**Sample-size estimation**

- You should state whether an appropriate sample size was computed when the study was being designed
- You should state the statistical method of sample size computation and any required assumptions
- If no explicit power analysis was used, you should describe how you decided what sample (replicate) size (number) to use

Please outline where this information can be found within the submission (e.g., sections or figure legends), or explain why this information doesn’t apply to your submission:

*State whether an appropriate sample size was computed when the study was being designed*

We used 156 flies to test each receptor type, with three groups (Gal4 control, UAS control, and test) for a total N ≈ 52, 52, 52 flies per experiment. This was selected from a power table to give a power of >70% for an effect size of 0.5 standard deviations.

*State the statistical method of sample size computation and any required assumptions*

A power table was inspected. We assumed that effect sizes <0.5 SD would be less interesting valence properties.

*If no explicit power analysis was used, describe how you decided what sample (replicate) size (number) to use.*

N/A

**Replicates**

- You should report how often each experiment was performed
- You should include a definition of biological versus technical replication
- The data obtained should be provided and sufficient information should be provided to indicate the number of independent biological and/or technical replicates
- If you encountered any outliers, you should describe how these were handled
- Criteria for exclusion/inclusion of data should be clearly stated
- High-throughput sequence data should be uploaded before submission, with a private link for reviewers provided (these are available from both GEO and ArrayExpress)

Please outline where this information can be found within the submission (e.g., sections or figure legends), or explain why this information doesn’t apply to your submission:

*Report how often each experiment was performed*

Experiments were performed once per each condition. Information provided in the Methods section.

*Include a definition of biological versus technical replication*

Technical replicates were not used for averaging in the statistical analysis. Information provided in the Methods section.

*The data obtained are provided…*

At the Zenodo repository cited in the manuscript. Link provided in the Methods section.

*Sufficient information is provided to indicate the number of independent biological and/or technical replicates*

Yes. Information provided in the Methods and Results sections.

*If you encountered any outliers, describe how these were handled*

Any outliers were handled as an integral part of the data, i.e. were not given special treatment.

*Criteria for exclusion/inclusion of data should be clearly stated*

All data were included, no data were given special exclusion.

*High-throughput sequence data should be uploaded before submission, with a private link for reviewers provided (these are available from both GEO and ArrayExpress)*

N/A

**Statistical reporting**

- Statistical analysis methods should be described and justified
- Raw data should be presented in figures whenever informative to do so (typically when N per group is less than 10)
- For each experiment, you should identify the statistical tests used, exact values of N, definitions of center, methods of multiple test correction, and dispersion and precision measures (e.g., mean, median, SD, SEM, confidence intervals; and, for the major substantive results, a measure of effect size (e.g., Pearson's r, Cohen's d)
- Report exact p-values wherever possible alongside the summary statistics and 95% confidence intervals. These should be reported for all key questions and not only when the p-value is less than 0.05.

Please outline where this information can be found within the submission (e.g., sections or figure legends), or explain why this information doesn’t apply to your submission:

*Statistical analysis methods should be described and justified.*

Estimation methods were used throughout, described in detail in the Methods section, and specified in the code.

*Raw data should be presented in figures whenever informative to do so (typically when N per group is less than 10)*

Raw data are presented for critical control experiments, but the overall sample is too large to do this: the screen uses more than 15,000 animals, for which effect sizes are reported. This information is provided in the Methods and Results sections.

*For each experiment, you should identify the statistical tests used, exact values of N, definitions of center, methods of multiple test correction, and dispersion and precision measures (e.g., mean, median, SD, SEM, confidence intervals; and, for the major substantive results, a measure of effect size (e.g., Pearson's r, Cohen's d)*

We consider p-values to be misleading, so do not use them for interpretation and report them for legacy purposes only (i.e. to placate readers who expect them). We report effect sizes with their precision estimates throughout the Results section.

*Report exact p-values wherever possible alongside the summary statistics and 95% confidence intervals. These should be reported for all key questions and not only when the p-value is less than 0.05.*

P-values are reported for satisfying legacy requirements only.

(For large datasets, or papers with a very large number of statistical tests, you may upload a single table file with tests, Ns, etc., with reference to sections in the manuscript.)

**Group allocation**

- Indicate how samples were allocated into experimental groups (in the case of clinical studies, please specify allocation to treatment method); if randomization was used, please also state if restricted randomization was applied
- Indicate if masking was used during group allocation, data collection and/or data analysis

Please outline where this information can be found within the submission (e.g., sections or figure legends), or explain why this information doesn’t apply to your submission:

*Indicate how samples were allocated into experimental groups (in the case of clinical studies, please specify allocation to treatment method); if randomization was used, please also state if restricted randomization was applied.*

Samples were allocated based on genotype, which is the appropriate way for this type of experiment.

*Indicate if masking was used during group allocation, data collection and/or data analysis*

Masking was not used at any stage.

**Additional data files (“source data”)**

- We encourage you to upload relevant additional data files, such as numerical data that are represented as a graph in a figure, or as a summary table
- Where provided, these should be in the most useful format, and they can be uploaded as “Source data” files linked to a main figure or table
- Include model definition files including the full list of parameters used
- Include code used for data analysis (e.g., R, MatLab)
- Avoid stating that data files are “available upon request”

Please indicate the figures or tables for which source data files have been provided:

*We encourage you to upload relevant additional data files, such as numerical data that are represented as a graph in a figure, or as a summary table.*

The data has been uploaded to a Zenodo repository.

*Where provided, these should be in the most useful format, and they can be uploaded as “Source data” files linked to a main figure or table*

We have not linked Source data to a main figure or table, but all the data is available at the Zenodo repository.

*Include model definition files including the full list of parameters used*

Arbitrary parameters were only required for fitting prediction models with the scikit-learn library in Python. The parameters—regularization constant (C), polynomial degree, and epsilon—were optimized for linear and nonlinear models when necessary by an automated grid-search algorithm, where the performance of the parameters was measured using validation curves. The search algorithms, parameters tested, and the final parameters used in the analyses are available in the code published on GitHub.

*Include code used for data analysis (e.g., R, MatLab)*

Code is available on GitHub.

*Avoid stating that data files are “available upon request”*

Avoided, relevant data files are uploaded to Zenodo repository.
